# How do compulsory citizenship behaviors affect moral disengagement in organizations? Significance of anger toward the organization during the COVID-19 pandemic

**DOI:** 10.3389/fpsyg.2022.1038860

**Published:** 2022-11-25

**Authors:** Bora Yildiz, Harun Yildiz, Mustafa Ozbilgin

**Affiliations:** ^1^Faculty of Economics, Department of Management, Istanbul University, Istanbul, Turkey,; ^2^College of Business, Arts and Social Sciences, Brunel Business School, Organizations and People, Brunel University London, Uxbridge, United Kingdom; ^3^School of Business, Economics and Informatics, Department of Organizational Psychology, University of London-Birkbeck College, Bloomsbury, United Kingdom; ^4^Omer Seyfettin Faculty of Applied Sciences, Department of International Trade, Bandirma Onyedi Eylul University, Balikesir, Turkey

**Keywords:** anger toward organization, compulsory citizenship behaviors, moral disengagement, nurses, employee citizenship

## Abstract

**Background:**

With the COVID-19 pandemic, healthcare professionals, especially nurses, are confronted with an intensified workload. The literature on compulsory citizenship behaviors and their consequences is still far from explaining the cognitive and emotional mechanisms that underlie this relationship.

**Methods:**

Drawing on the resource depletion theory, we unpack the mechanism by which compulsory citizenship behaviors influence moral disengagement with the mediation effects of anger toward the organization. We are reporting a cross-sectional survey of nurses (*n* = 294) in private and public hospitals in Istanbul, Turkey. The data analysis involved structural equation modeling and Bayesian mediation.

**Results:**

The study revealed that compulsory citizenship behaviors positively influenced anger toward the organization and moral disengagement. Further, anger toward the organization mediates the link between compulsory citizenship behaviors and moral disengagement. Likewise, the Bayesian mediation analysis indicated that the proportion mediated (PM), which ensures a prediction of the extent to which the pathway explains the total effect through the mediation effect, was 33.74%.

**Conclusion:**

The findings show that exposure to compulsory citizenship behaviors lead to negative emotional (anger toward to organization) and cognitive (moral disengagement) consequences in nurses.

**Practical implications:**

Hospital managers should not force nurses to display discretionary work tasks outside their job descriptions. Providing an organizational milieu where voluntarily extra-role behaviors are encouraged may help reduce nurses’ moral disengagement and, in turn, ease their anger toward the organization.

## Introduction

In 2020, the number of nurses working in Turkey was 198.465 (Turkish Ministry of Health, 2020). The figure is inadequate compared with other countries in the Organization for Economic Co-operation and Development (OECD). The number of patients per nurse is four times higher than the OECD average (Turkey = 413 patients per nurse; OECD average = 102 patients per nurse; Turkish Ministry of Health, 2020). While these indicators draw attention to the excessive workload of nurses in Turkey (Yildiz et al., 2021), they also explain why managers and organizations may be forcing nurses to do jobs outside of their job descriptions. Further, data from the Turkish Ministry of Health show that patient examinations are in an increasing trend. For example, the annual number of examinations was 235 million in 2010, and the number of requests per person stood at 3.9. In 2016, the number of examinations was 340 million, and the number of requests per person was 4.3 (Public Hospitals Administration of Turkey, 2016). Although there is a general growth in the number of hospitals and personnel in Turkey, the measures taken to combat the intensification of work for nurses remain insufficient. Addressing staff shortages becomes even more urgent for policymakers in crisis conditions (Rasmussen et al., 2020; Turale et al., 2020). Extra-contractual work behaviors are imposed by organizations or management and are defined as compulsory citizenship behaviors (CCBs). Previous research indicates that nurses have found it increasingly hard to handle CCBs in extreme conditions such as the COVID-19 pandemic (Boztilki et al., 2021; Yildiz et al., 2022b).

The COVID-19 pandemic has rendered much conventional wisdom about healthcare workers’ workload (Yildiz et al., 2021; Boztilki et al., 2021), professionalism (Cici and Yilmazel, 2021), job satisfaction (Said and El-Shafei, 2021), work engagement (Giménez-Espert et al., 2020), motivation (Sperling, 2021), and psychological resilience questionable (Bozdağ and Ergün, 2021). For example, a recent meta-analytic study found that the relationship between work engagement and job satisfaction, which was positively and highly correlated before COVID-19, turned negative during the COVID-19 process (Yildiz et al., 2022b). Another study revealed that health workers, especially nurses, experienced a moral injury during the COVID-19 process (Hossain and Clatty, 2021). Nurses have experienced significant psychological traumas, especially in the middle of the pandemic, due to the ever-increasing number of patients, deaths, and working hours. The surging number of deaths during the pandemic made them feel inadequate and responsible (Hossain and Clatty, 2021). In addition, nurses could not spare time for their families and had to limit their social lives due to the risk of disease transmission (Hossain and Clatty, 2021; Yayla and Eskici İlgin, 2021). When CCBs were added to these dire conditions, nurses started feeling angry toward their organizations, which failed to empathize with nurses, increased their stress levels, and reduced their psychological resilience (Huang et al., 2020; Hossain and Clatty, 2021; Huerta-González et al., 2021). In other words, anger toward the organization is not only caused by the conditions of work during the COVID-19 pandemic but also by CCBs (Che, 2015).

Although the literature on CCBs continues to grow, studies on emotional responses to CCBs, especially feelings of anger, are limited (Che, 2015). Jameton (1993) asserts that anger or frustration could lead to moral disengagement, defined as the deactivation of self-regulatory mechanisms in the ethical decision-making process (Bandura, 1986, 1999) and justifying unethical or immoral actions as if it is normal (Zhang et al., 2019). Supporting this notion, some studies considered the decisive role of emotions in moral decision-making mechanisms (Muraven and Baumeister, 2000; Sreeshakthy and Preethi, 2016; Fida et al., 2018) and explored the underlying role of anger in the literature will contribute to the literature in understanding how moral disengagement occurs because of CCBs. This study extends the extant literature by examining the effects of the challenging working conditions of COVID-19 on nurses and their CCBs on their emotions and moral decision-making mechanisms. In this context, the aims of this study are as follows:

1. To determine the CCBs, anger, and moral disengagement levels of nurses during the COVID-19 pandemic.
2. To determine how CCBs affected moral disengagement during the COVID-19 pandemic.
3. To test the mediator role of anger in the CCBs-moral disengagement relationship based on the resource depletion theory

## Literature review

### Compulsory citizenship behaviors

Studies on job performance have highlighted the positive role of organizational citizenship behavior (OCB; Yildiz, 2019). While job performance measures the employee’s capacity to perform their assigned duties at work, OCB refers to the employee’s involvement in voluntary activities (Vigoda-Gadot, 2007; Mugayar-Baldocchi, 2021). Extra-role behaviors, which are discretionary, contribute to organizations’ efficient and effective functioning by creating healthier and collegiate work environments (Vigoda-Gadot, 2006). Encouraging extra-role behaviors is an important strategy for organizations to achieve their goals (Yildiz and Yildiz, 2015). These behaviors will increase productivity and contribute to a more peaceful and productive climate in the workplace (Zhuang, 2021). Therefore, employees who see discretionary behaviors are more likely to display similar behaviors (Shaw et al., 2020). Enforcing behaviors that are supposed to be voluntary transforms such extra-role behaviors into in-role behaviors (Youn et al., 2017; Yildiz and Elibol, 2021). In this context, CCBs appear as the dark side of OCB, as CCBs are enforced while OCB remains voluntary. Research shows that CCBs negatively affect employee productivity (Irby, 2021). Employees who feel pressured to exhibit CCBs tend to show lower levels of creativity and creative self-efficacy (He et al., 2020), organizational identification, organizational citizenship behavior, and perceived interactional justice (Zhao et al., 2014). The studies also showed that employees who are exposed to CCBs suffer from higher levels of work–family conflict (Pradhan and Gupta, 2021), show more social loafing and turnover intention (Yildiz and Elibol, 2021), psychological withdrawal (Bashir et al., 2019), anger toward organization (Che, 2015), and moral disengagement (He et al., 2019).

### Anger toward organizations

Lee and Allen (2002) remarked that emotion and cognition are two critical action drivers. Spector and Fox (2002) supported this notion that organizations are complex environments that cause strong emotions. Anger as an emotion is described as “a syndrome of relatively specific feelings, cognitions, and physiological reactions linked associatively with an urge to injure some target” (Berkowitz and Harmon-Jones, 2004, p. 108). Anger can also be seen as a defense mechanism that is felt intensely in the emotional spectrum and occurs as a response to situations or events experienced by the person (Robbins and Judge, 2013). For example, employees work overtime in response to pressure from their managers, and as a result, they may develop negative feelings toward their managers. If these feelings turn into a more robust form, anger is felt, and the role of anger here is a defense mechanism (Masango, 2004). For example, employees may feel angry when they perceive organizational injustice against themselves (Zhuang, 2021). It is natural for employees to feel anger toward their organizations when their job expectations and personal goals are not met for various reasons (Gibson and Callister, 2010). Although it is used as a defense mechanism, anger emotion has a feature that impairs the employee’s ability to work effectively (Robbins and Judge, 2013). Anger toward an organization positively affects cyberloafing and counter-productive workplace behaviors (Zhang et al., 2019; Zhuang, 2021).

Additionally, anger and hostility appear as positive and significant drivers of moral disengagement (Rubio-Garay et al., 2016). The use of moral disengagement as a strategy to overcome an emotion such as anger or sadness (Hystad et al., 2014; Fida et al., 2015) may have devastating consequences such as unethical work behaviors or counter-productive workplace behaviors (Moore et al., 2012; Samnani et al., 2014), for the organization. Therefore, managers are often expected to deal with negative emotions proactively.

### Moral disengagement

Moral disengagement (MD) is based on Bandura’s theory of moral agency (Bandura, 1986). MD helps explain why individuals may behave in immoral or unethical ways (D’Errico and Paciello, 2018). MD is a precursor to harmful tendencies to break the rules and justify unethical acts. MD also accounts for why and how individuals justify their unethical behaviors. MD among staff often does not serve organizational interests. As a negative coping strategy (Hystad et al., 2014; Fida et al., 2015), MD refers to the deactivation of self-regulatory mechanisms in the ethical decision-making process (Bandura, 1986, 1999). Employees justify their unethical and immoral actions using moral disengagement mechanisms (Zhang et al., 2019). Detert et al. (2008, p. 375) stated that “hrough moral disengagement, individuals are freed from the self-sanctions and the accompanying guilt that would ensue when behavior violates internal standards, and they are therefore more likely to make unethical decisions.” Past studies showed that moral disengagement is related to unfavorable outcomes, such as counterproductive work behaviors (Hystad et al., 2014), cyberloafing (Zhang et al., 2019), sabotage toward customers (Huang et al., 2019), aggressive behaviors (Gini et al., 2014), bullying (Pozzoli et al., 2012), unethical decision-making (Detert et al., 2008; Moore, 2008), and social loafing (Hou et al., 2021). In this study, MD leads to harmful consequences when negative emotions emanating from being coerced into extra-role behaviors are not combated. In this study, MD is considered as one of the possible outcomes that may arise when negative emotions emanating from being coerced into extra-role behaviors are not combated.

## Theoretical background and hypothesis development

According to Hackman and Oldham (1980), the nursing profession has high task significance, skill variety, and task identity, which leads employees to experience work more meaningfully. Therefore, nurses display high intrinsic motivation (Toode et al., 2015). They engage in voluntary behaviors outside their roles (Wibowo and Mochklas, 2020). For example, a nurse may skip lunch to help their friend who is on duty. Such altruistic behaviors are common in this profession, and many nurses engage in similar discretionary behaviors (Bahrami et al., 2016). However, managers may sometimes view these voluntary behaviors as duties and enforce these behaviors. Considering uneven relations of power within the organization, it is difficult for employees to object to these expectations (Jervis, 2002; Mugayar-Baldocchi, 2021). In addition to the above arguments, healthcare workers suffered precarious working conditions due to ineffective management of the pandemic during the COVID-19 process (Hossain and Clatty, 2021; Greenhalgh et al., 2022). This situation has made CCBs a common imposition for nurses across many national healthcare systems. It was not always possible for the nurses to express their dissatisfaction or complaints arising from CCBs during the COVID-19 pandemic.

Organizational scholars and practitioners have recently devoted significant attention to investigating the antecedents and consequences of CCBs (Zhao et al., 2014; He et al., 2018; Shu et al., 2018; Unaldi Baydin et al., 2020; Yildiz and Elibol, 2021). Employees naturally have some reactions to managers or organizations that expose them to forcible OCB, and anger emotion is one of them (Che, 2015). As previously expressed, anger as an emotion is described as “a syndrome of relatively specific feelings, cognitions, and physiological reactions linked associatively with an urge to injure some target” (Berkowitz & Hamon-Jones, 2004, p. 108). From the resource depletion theory (Kong and Drew, 2016), if extra-role behaviors are reluctantly fulfilled with violent actions of nursing managers or powerful others, they can cause a reduction and loss of resources for nurses. Therefore, employees are emotionally activated (Vigoda-Gadot, 2007; Fida et al., 2015; Gou et al., 2022) and feel anger toward the organization (Che, 2015). Anger and compulsivity are also some of the consequences of COVID-19 (Huang et al., 2020; Huerta-González et al., 2021). Supporting this notion, COVID-19 related stress causes some negative results, such as anger, anxiety, insomnia, and depression (Huang et al., 2020; Zhang et al., 2020). Considering the stressful, tiring, and challenging conditions of COVID-19 and the stress created by the extra burdens of CCBs on employees, nurses can develop anger toward the organizations they work for. In this context, the first hypothesis of the research is as follows:

*Hypothesis 1:* Compulsory citizenship behaviors are likely to lead to anger.

As previously mentioned, nursing is a profession that requires a high level of intrinsic motivation and is performed by people with this motivation (Toode et al., 2015). It is natural for individuals with this characteristic to have high expectations for their jobs and organizations (Bodur and İnfal, 2015). However, the gap between employees’ expectations and reality can cause them to feel angry (Liu et al., 2015). Anger is one of the six basic emotions, and it is an emotion that is felt most intensely on the emotional scale, and its reactions are more substantial than other emotions (Robbins and Judge, 2013). Nurses have reportedly felt powerless and inadequate during COVID-19 (Ju-Hong et al., 2020; Joo and Liu, 2021), and the CCBs they were exposed to may cause nurses to remain inactive to keep their jobs (Yildiz and Elibol, 2021). When they experience organizational obstacles or conflicts about their values, they experience anger, frustration, and anxiety (Jameton, 1993; Corley, 2002). According to this view, unpleasant work environments cause anger toward organizations. Also, nurses may feel angry toward their organization when their fair and decent work expectations are not met. The anger and hostility experienced may justify their moral disengagement (Rubio-Garay et al., 2016).

Moral disengagement, as a negative coping strategy (Hystad et al., 2014; Fida et al., 2015), refers to the deactivation of self-regulatory mechanisms in the ethical decision-making process (Bandura, 1986, 1999). Anger is one of the antecedents of moral disengagement (Fida et al., 2018) that plays a critical role in laying the groundwork for adverse consequences such as aggressive behaviors (Fontaine et al., 2014; Rubio-Garay et al., 2016). Previous studies found that anger plays a distorting role in the self-regulatory mechanism of the moral evaluation process that results in moral disengagement (Caprara et al., 2013). By activating moral disengagement, nurses try to overcome emotions and feelings that are the product of the stress mentioned above (Zhang et al., 2019). Zhang et al. (2019) revealed a positive relationship between anger toward an organization and moral disengagement. The second research hypothesis developed based on the above explanations is as follows:

*Hypothesis 2:* Anger is likely to lead to moral disengagement.

The nursing profession has a high-stress level due to its focus on eliminating mistakes that can damage lives (Sun et al., 2020). In addition to the potential stress caused by the characteristics of the job, intensification and precarious conditions cause nurses to suffer from high workloads and stress (Khademi et al., 2015; Youn et al., 2017; Hardiyono et al., 2020). During the COVID-19 pandemic, this workload has peaked, and the balance between work and family has been jeopardized (Yildiz et al., 2021; Boztilki et al., 2021). In this process, it has become necessary to exhibit CCBs, such as working overtime without pay. During the COVID-19 pandemic, nurses have consciously performed these obligatory behaviors for public health and saving the lives of others because there were no proactive social policies to manage their excessive workload (Lucchini et al., 2020). However, the tolerance level of the nurses on this issue has also become fragile (Turale et al., 2020).

Lee and Allen (2002) pointed out that emotion and cognitions are crucial drivers of actions. Because CCBs are out of the expectations of nurses toward their job, frustrated nurses can develop some cognitive solutions to overcome their frustration. From the social cognitive theory perspective (Bandura, 1999), it is usual for the individual to nurture anger and similar emotions in response to the stimuli (e.g., frustration) the environmental factors (Arda and Yildiz, 2019). On the other hand, from the resource depletion theory perspective (Kong and Drew, 2016), it is usual for the individual to develop cognitive strategies to reduce the stress factors he/she is exposed to and to protect his/her positive resources. When these two theories coincide, it becomes possible for the individual to develop immoral solutions, such as moral disengagement, that will minimize the damage he suffers with his/her cognitive mechanisms when he cannot find a solution. To draw attention to the harmful effects of CCBs on employees, a recent study noted that “CCBs will generate negative emotions and cause retaliation tendencies” (He et al., 2019: p. 263). Therefore, we tend to believe that when nurses are compelled to perform CCBs outside of their duties, they may suffer moral and psychological distress due to these behaviors outside of their free will. Moreover, employees may feel entitled to act without moral considerations (morally free) on the grounds of these distresses (He et al., 2019). In other words, the perception that the organization or managers harm them will disable the self-regulation mechanisms of the employees. It will pave the way for their moral disengagement (Lee et al., 2016). Such moral disengagement may harm the organization (e.g., counterproductive workplace behaviors, sabotage behaviors, silence, and unethical workplace behaviors) (Vigoda-Gadot, 2006, 2007; Shao et al., 2008; Moore et al., 2012; Heald, 2017; Thrasher et al., 2020; Zhuang, 2021). Given our arguments above, we posit that:

*Hypothesis 3:* Compulsory citizenship behaviors lead to moral disengagement.

**Figure 1:**
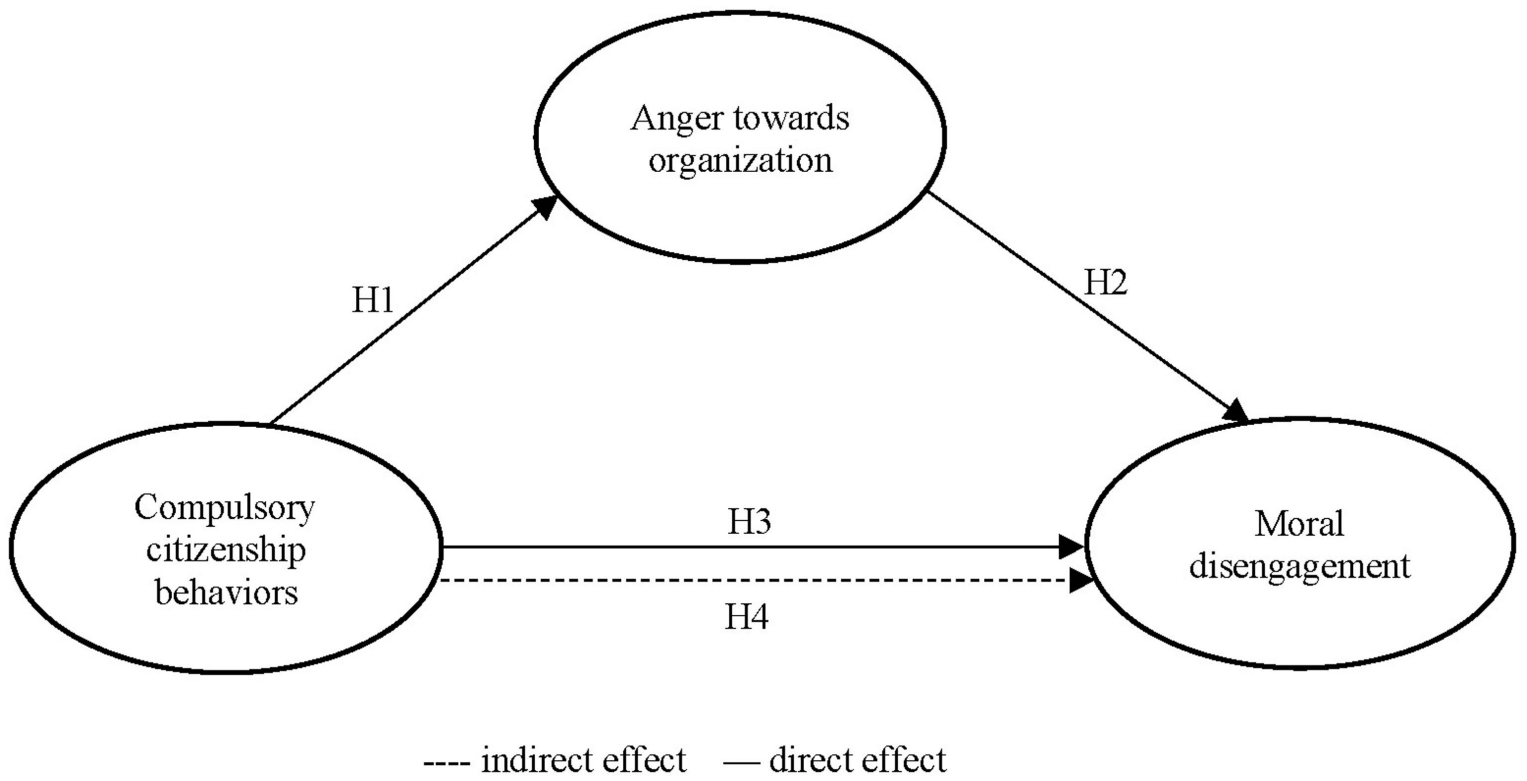
Theoretical model.

According to the resource depletion theory, individuals’ resources (Muraven et al., 1998; Muraven and Baumeister, 2000; Kong and Drew, 2016) can be depleted by acts that require overwhelming self-initiated or various situational demands. Because individuals’ self-regulation capacity is finite, prolonged self-regulation decreases this resource and consumes it after that (Selart and Johansen, 2011). Therefore, once nurses involuntarily exhibit extra behaviors due to violent actions (CCBs) by nursing managers and powerful others, they are emotionally activated (Fida et al., 2018) and may feel anger toward the organization. Negative emotions such as anger trigger moral disengagement because of a weakened moral self-regulation system (Barnes et al., 2011; Christian and Ellis, 2011; Fida et al., 2015). Moral disengagement is characterized as a flexible cognitive orientation within employees and activated to be depleted of resources in specific circumstances like CCBs (Hystad et al., 2014; Gou et al., 2022). CCBs make nurses inclined to moral disengagement by consuming nurses’ capacity for self-regulation (Selart and Johansen, 2011). Self-regulatory abilities are also responsible for impaired emotional mechanisms (Muraven et al., 1998; Schmeichel and Baumeister, 2004) because negative emotions like anger, sadness, and hostility originated from impairments in these capabilities (Muraven and Baumeister, 2000; Christian and Ellis, 2011). As mentioned above, “CCBs will generate negative emotions and cause retaliation tendencies” (He et al., 2019: p. 263). Considering the above explanations, we believe that anger toward the organization and moral leniency will be one of the emotional and cognitive retaliation tendencies against the psychological and moral distress caused by CCBs. To give answer to research calls on potential mediators that can affect the direct link between CCBs and their consequences (Vigoda-Gadot, 2006; Yildiz and Yildiz, 2016; Fida et al., 2018), this study explores the relationship between CCBs, and moral disengagement utilizing resource depletion theory and uses anger toward organization as a mediator in the proposed link (see Figure 1). Given our arguments above, we posit that:

*Hypothesis 4:* Anger mediate the direct relationship between compulsory citizenship behaviors and moral disengagement.

## Materials and methods

*R* statistical environment (R Core Team, 2020) was utilized for analyses of the data in this study. To analyze the data following packages were used “lavaan” (Rosseel, 2012), “psych” (Revelle, 2022), “Performance Analytics” (Peterson and Carl, 2020), “BayesFactor” (Morey and Rouder, 2022), “haven” (Wickham et al., 2022), “performance” (Lüdecke Lüdecke et al., 2021), and “parameters” (Lüdecke et al., 2020).

Firstly, descriptive analyses were conducted. Then, an outlier analysis was conducted to detect and clean the outliers from the dataset. Explanatory and confirmatory analysis was conducted to test construct validity. Cronbach’s alpha test was conducted to test scale reliabilities. To determine the relationships among the study variables, correlation analysis was conducted. Lastly, covariance-based structural equation modeling and Bayesian mediation analyses were performed to test research hypotheses.

### Participants and procedure

The unit of analysis of this study is individuals. Accordingly, the participants of this study are nurses working at private and public hospitals in Istanbul, Turkey. The snowball sampling method was used to collect data. The snowball sampling method is widely used for collecting data on sensitive issues where the research focus is very small/specific or where respondents are hesitant to respond to statements related to the research topic (Browne, 2005). Therefore, this type of research is not only a data collection method but also plays an important role in protecting the anonymity of respondents in terms of not experiencing stress, fear of being fired fear of disclosure, and social pressure. Since CCBs emerge as a result of the pressures of cultural, managerial, or powerful others on employees (Vigoda-Gadot, 2006, 2007), and since the feeling of anger toward the organization is not welcomed by the managers in collectivist cultures (Chen et al., 2018, 2020), it is assumed that employees will not give a sincere or honest answer in the data collection phase to avoid the fear of dismissal, mobbing, and abusive behaviors of the managers. Also, in collectivist cultures, as in Turkey (Hofstede, 2022), an individual’s immoral tendencies and behaviors can be shaped according to other people in society (Romera et al., 2022). Therefore, the participants may hide their moral disengagement tendencies to avoid losing their social comfort areas in society. As Browne (2005) suggested, social networks and focus groups play a crucial role in conducting this type of research. In this context, the researchers reached nurses through their contacts. Because of the ongoing Covid-19 pandemic, the purpose and scope of the study were informed to fifteen nurses in a series of online meetings. To be a participant in the study, the inclusion criteria were set as follows:

1. Being employed in a public or private hospital.
2. Occupying non-managerial positions.
3. Having served in their present department for at least 1 year.
4. Being in direct contact with at least one manager or supervisor.

Afterward, an online survey link was shared with these nurses to send to their colleagues. A total of 322 valid surveys were returned, of which 28 were determined to be outliers and excluded from the study by using Mahalanobis distance (Penny, 1996). After excluding the outliers, the final sample size consists of 294 valid questionnaires. Most of the nurses (86%) were females and married (74.1%). The age of participants varied between 21 and 54, with a mean of 32.20 (SD = 11.06). The majority had received higher education (45% bachelor’s degree holders, 19% postgraduate degree holders). 221 (75.2%) of the respondents work in public hospitals (see Table 1).

**Table 1:** Demographic characteristics of the sample (*n* = 294).

Demographics	*n*	*%*
*Gender*		
Female	253	86
Male	41	14
*Education*		
Postgraduate	54	18.3
Graduate	53	18
Vocational school	132	44.9
High school	55	18.8
*Age* (= 32.20 ± 11.06)		
*Sector*		
Public	221	75.2
Private	73	24.8
*Marital status*		
Married	218	74.1
Single	76	25.9
Total	294	100

### Measures

Data were obtained using an online survey platform, on which participants could complete the survey on a five-point Likert scale based on a five-point scale anchored at 5 (strongly agree) and 1 (strongly disagree). The survey had three sections and 16 items (see Appendix). In the first part, socio-demographic factors, such as gender, age, marital status, level of education, and job tenure were included in the study. The other parts contain the three different measurement tools with a Likert type format. Although there are many scales in the related literature to measure the constructs in the research, the reason for using these scales detailed below is that the scales were applied to employees and organizational settings, and they are reliable and valid scales.

#### Compulsory citizenship behaviors

The one-dimensional and five-item scale developed by Vigoda-Gadot (2007) and adopted into Turkish by Yildiz (2022) was used for CCBs. A sample item of the scale is “The management in this hospital puts pressure on nurses to engage in extra-role work activities beyond their formal job tasks.” The main reason for using this scale is that an ongoing meta-analytic study on 42 publications on CCBs (Yildiz et al., 2022a) has demonstrated that the scale is a reliable and valid measurement tool. The average Cronbach alpha coefficient in the mentioned 42 studies was a = 0.86 ±. 07 (a_min_ = 0.65, a_max_ = 0.96).

#### Moral disengagement

The one-dimensional and eight-item scale developed by Moore et al. (2012) was utilized to measure MD. The scale back translated to Turkish by following guideline of Brislin (1970). An example item of the scale is “People should not be held accountable for doing questionable things when they were just doing what an authority figure told them to do.” Although there are many measurement tools related to MD, the reliability coefficient of this eight-item and one-dimensional scale in the original study was calculated as a = 0.80 (Moore et al., 2012). When compared with the 16-item (a = 0.88) and 24-item (a = 0.90) versions, the eight-item scale was accepted as reliable (Zheng et al., 2019) because the reliability coefficient of the scale was higher than a = 0.70 value (Cronbach, 1951).

#### Anger toward organization

The one-dimensional and three-item scale developed by Fredrickson et al. (2003) was operationalized to gage anger toward organization (ATO). The scale back translated to Turkish by following guideline of Brislin (1970). A sample item of the measure is “I feel angry toward my organization.” Previous studies showed that the reliability coefficient of the scale is well above the acceptable values, that is a = 0.91 (Mitchell et al., 2015).

### Validity and reliability

The measurement model of three constructs was tested by confirmatory factor analysis (CFA). When the fit indices of the initial model are evaluated, the model fit indices are as follows; [*x*^2^ (314.088)/df (101) = 3.110, GFI = 0.88, AGFI = 0.85, NFI = 0.91, CFI = 0.94, TLI = 0.93, SRMR = 0.049, RMSEA = 0.085; *p* < 0.05 [90% CI = 0.07–0.10]]. When the modification indices were checked, it was found that there was a high correlation between the error terms of the cc1-cc2, ccb-ccb3, and md4-md8 items. The high correlation between the error terms is due to systematic errors (Kline, 2015) arising from the characteristics of the items or the respondents (Byrne, 2001). If the wording of the items is different, it is not recommended to improve the model by correlating the error terms (Byrne, 2001). On the other hand, if their contents are the same and correlated error terms, they are within the same construct and thus, the modifications cause minor improvements instead of major changes (Khanna et al., 2012). Accordingly, to improve the model fit, the error terms of the specified items are correlated. The results of CFA demonstrated that relatively good model–data fit in general [*x*^2^ (145.979)/df (97) = 1.504, GFI = 0.95, AGFI = 0.93, NFI = 0.96, CFI = 0.99, TLI = 0.99, SRMR = 0.042, RMSEA = 0.035; *p* < 0.05 [90% CI = 0.065–0.150]] to the fit criteria recommended by past researchers (Anderson and Gerbing, 1984; Schermelleh-Engel et al., 2003; Schumacker and Lomax, 2004). Besides, we calculated the convergent and discriminant validity of each construct, average variance extracted (AVE), and Fornell and Larcker’s (F-L) criterion were assessed (Kaya et al., 2020). All the AVE values for the convergent validity were above the minimum required value of 0.50. The F-L estimates determined for the discriminant validity were satisfactory and the F-L values of all constructs were higher than their correlations with other variables. Regarding the reliability of each construct, we evaluated both Cronbach’s (α) coefficient and composite reliability (CR) estimates. The alpha coefficients of CCBs of Cronbach (1951), anger toward organization, and moral disengagement are as follows; α = 0.91, α = 0.89, and α = 0.97, respectively. In addition to α coefficient, the CR values were above the minimum standard of 0.70 (Hair et al., 2013). Consequently, the available results met the required standards for validity and reliability. All results can be seen in Table 2.

**Table 2:** Means, standard deviations, and correlations with confidence intervals (*n* = 294).

Variable	*M*	*SD*	CR	AVE	1	2	3	4	5	6	7	8
1. Gender	–	–	–	–	1							
2. Sector	—	–	–	–	0.12^*^	1						
3. Marital S.	—	–	–	–	0.12^*^	−0.06	1					
4. Education	2.64	0.99	–	–	−0.06	−0.05	0.05	1				
5. Age	32.2	11.1	–-	–	0.02	−0.07	0.51^**^	0.15^*^	1			
6. CCBs	2.65	1.15	0.90	0.65	0.04	0.03	0.03	0.19^**^	−0.06	(0.91)		
7. ATO	2.20	1.06	0.89	0.50	0.07	−0.05	0.04	0.14^*^	0.06	0.40^**^	(0.97)	
8. MD	1.77	0.78	0.87	0.92	0.12^*^	−0.01	0.01	−0.03	−0.08	0.21^**^	0.23^**^	(0.89)

Gender: 0 = Female, 1 = Male; Sector: 0 = Public, 1 = Private; Marital status: 0 = Single, 1 = Maried; CCBs, compulsory citizenship behaviors; ATO, anger toward organization; and MD, moral disengagement. M and SD symbolize mean and standard deviation, respectively. CR, composite reliability; AVE, average variance extracted. ^*^ indicates *p* < 0.05. ^**^ represents *p* < 0.01. Cronbach’s Alpha values are represented in parentheses.

### Common method bias

Harman’s (1979) one-factor method was used to test whether there is a common method bias (CMB) since the data regarding the variables within the scope of the research are cross-sectional data (Podsakoff et al., 2003). As a result of the exploratory factor analysis (EFA) in which all questions were included, it was concluded that each item was loaded on its own construct and the total variance explained was 70% (moral disengagement 28.9%, compulsory citizenship behaviors 23.4%, and anger toward to organization 17.8%). Additionally, one more EFA was performed without choosing any rotation method and by selecting only a single factor. It was determined that the amount of variance explained by a single general factor, including all items, was 36.5%, which is lower than 50%. This result indicates that there was no CMB in this study. In sum, we can assert that the findings are not affected by the CMB problem.

### Bayesian mediation vs. frequentist mediation

The hypothesized research model was tested with frequentist (Baron and Kenny, 1986; MacKinnon et al., 2002; Hayes, 2009) and Bayesian estimation. Conceptually Bayesian method is like frequentist mediation models calculated using Maximum Likelihood (Milfont and Sibley, 2016). However, the Bayesian mediation analysis has several advantages over the classical or frequentist approach (Yuan and MacKinnon, 2009). First, it provides a more robust statistical analysis, especially in small samples. Second, it combines prior and posterior knowledge when assessing the mediating effect, and lastly, it is more straightforward compared to the frequentist approach.

Bayesian estimates are comparatively more robust and interpretable (Milfont and Sibley, 2016). In this respect, the classical frequentist approach has some limitations. One of these limitations is that the indirect effect does not have a parametric distribution (MacKinnon et al., 2002). To overcome this problem, confidence intervals are used (Sobel, 1982), but the distribution of the indirect effect is still not symmetrically distributed (MacKinnon and Dwyer, 1993). Therefore, interpreting indirect effects is yet problematic in these methods. To cope with this problem, the bootstrap method (Hayes, 2009) and confidence intervals were suggested by past researchers (MacKinnon et al., 2002). However, they still have their limitations because these solutions are based on fixed-parameter estimation. In contrast, the Bayesian mediation analysis assesses parameters as random variables. For this reason, the Bayesian mediation analysis is more natural than the frequentist approach (Gelman and Hill, 2006). According to Yuan and MacKinnon (2009), the Bayesian method is expressed exactly as follows (p. 3): “All knowledge and uncertainty about unknown parameters are measured by probabilities. In contrast, conventional (or frequentist) statistical inference treats unknown parameters as unknown fixed values.”

### Ethics considerations

The ethical approval for this study was received from the Committee on Ethics in Research on Humans of X University (Approval number: 2021–142).

## Results

### Correlation analysis

Pearson correlation analysis demonstrated that the correlation results are as follows (see Table 2): Gender is positively associated with moral disengagement (*r* = 0.12; *p* < 0.05), marital status (*r* = 0.12; *p* < 0.05), and sector (*r* = 0.12; *p* < 0.05). Age positively correlated with marital status (*r* = 0.51; *p* < 0.01). CCBs positively associated with anger toward organization (*r* = 0.40; *p* < 0.01), and moral disengagement (*r* = 0.21; *p* < 0.01). Anger toward organizations positively related with moral disengagement (*r* = 0.23; *p* < 0.01).

### Frequentist (non-Bayesian) mediation analysis

**Figure 2:**
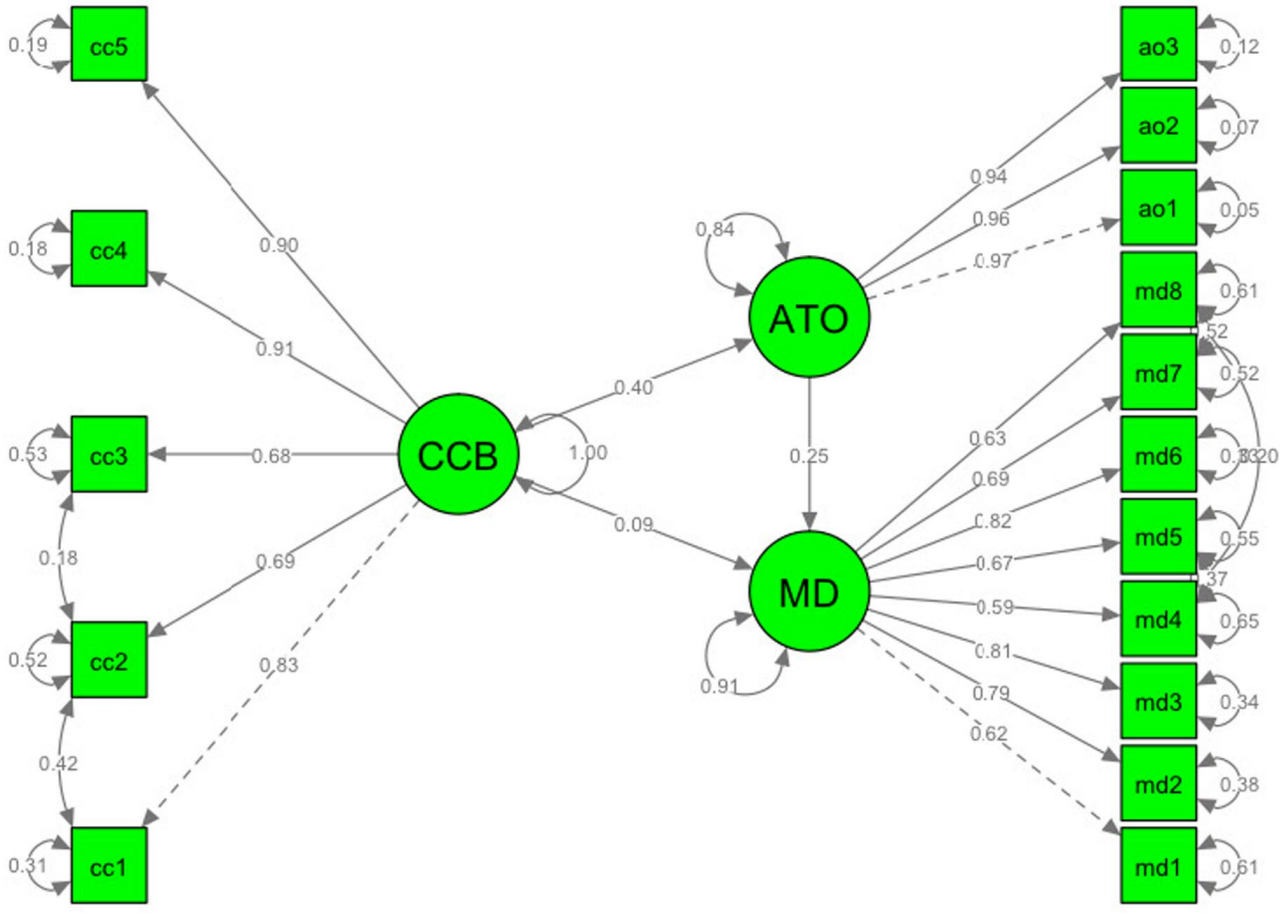
Path model with factor loadings.

A covariance-based structural equation model (CB-SEM) was used to validate the model. As seen from Table 3; Figure 2, compulsory citizenship behaviors positively affected both anger toward the organization and moral disengagement. Thus, Hypotheses 1 and 3 were supported. The study further indicated that anger toward the organization had a positive effect on moral disengagement. So, Hypothesis 2 was supported.

**Table 3:** CB-SEM path coefficients and Bootstrap analysis of the mediating effect.

CB-SEM path	Unstandardized path coefficient	Standardized path coefficient	S.E.	C.R	*p*
CCBs →ATO (path a)	0.40	0.40	0.058	6.764	0.000^***^
ATO → MD (path b)	0.17	0.25	0.046	3.583	0.000^***^
CCBs → MD (direct)	0.13	0.21	0.040	3.272	0.001^**^
CCBs → MD (path c’ indirect)	0.06	0.10	0.045	1.386	0.166 ns.
**Bootstrap analysis of the mediating effect**
**Structural path**				**Indirect effects**	**Lower bound**	**Upper bounds**	
CCBs → ATO → MD				0.05^*^	0.01	0.09	
Monte Carlo power analysis for indirect effect (n = 294)				0.98			

Bootstrap analysis of the mediating effect.

^*^ indicates *p* < 0.05, ^**^ represents *p* < 0.01, and ^***^ is *p* < 0.001.

**Figure 3:**
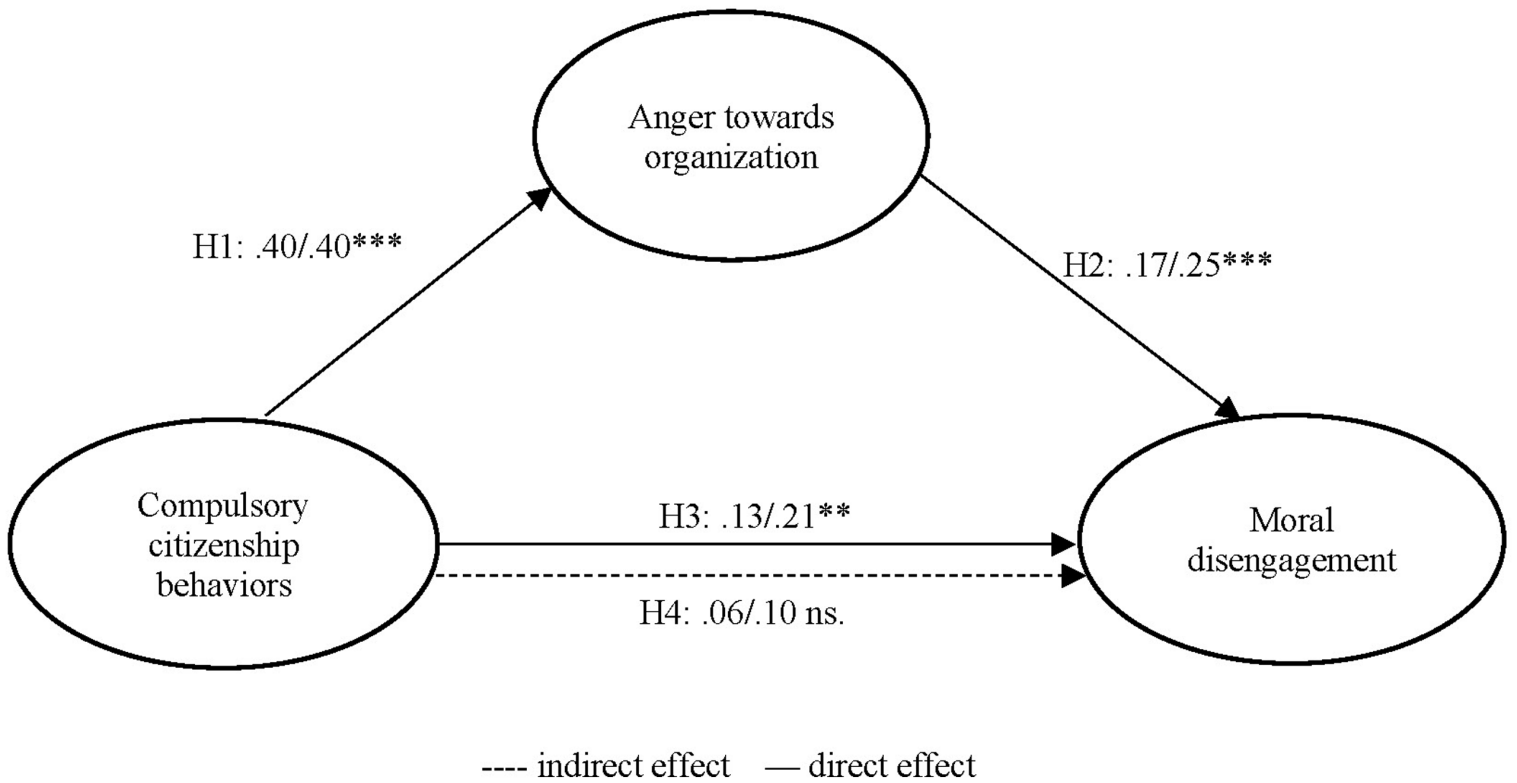
Unstandardized/standardized estimates of covariance-based structural equation model (CB-SEM). ns. indicates not significant (*p* > 0.05), ** represents *p* < 0.01, and *** represents *p* < 0.001.

To test the mediation effect of anger toward the organization, Bootstrap method was performed. As shown in Table 3; Figure 3, the indirect effect of compulsory citizenship behaviors on moral disengagement through moral disengagement was 0.05 with a bias-corrected and accelerated confidence interval (*BCa CI*) of [0.01–0.09] at the 95% level, and *BCa CI* did not include 0. Thus, Hypothesis 4 was supported. Accordingly, the indirect effect was found as a full mediation effect. Further, the statistical power of the mediation effect was determined as.98 by using Monte Carlo power analysis (Schoemann et al., 2017).

### Bayesian mediation analysis

The result of the Bayesian regression analysis was shown in Table 4. In accordance with this, average direct effect (0.10), average causal mediation effect (0.05), and total effect (0.14) were determined as positive and significant with confidence interval (CI) of at the 95% level. In summary, the results of the Bayesian mediation analysis are consistent with the well-known frequentist approach.

**Table 4:** Results of Bayesian mediation analysis.

Effects	Estimate	Quasi-Bayesian confidence intervals		*p*
		CI lower 95%	CI upper 95%	
Indirect effect (ACME)	0.0482	0.01	0.09	0.004^**^
Direct effect (ADE)	0.0955	0.02	0.18	0.022^*^
Total effect	0.1437	0.07	0.22	0.002^**^
Proportion mediated (PM)	0.3374	0.09	0.77	0.006^**^

ATO, anger toward organization; CCBs, compulsory citizenship behaviors; MD, moral disengagement; ADE, average direct effects; ACME, average causal mediating effect; and CI, confidence interval. * indicates *p* < 0.05 and ** represents *p* < 0.01.

On the other hand, proportion mediated (PM) is the ratio of total effect to natural indirect effect (VanderWeele, 2013; Ananth, 2019). Considering this view, the amount of PM ensures a prediction of “the extent to which the total effect is explained through the mediating variable” (Ananth, 2019, p. 983). Hence, the PM is a convenient measure in the Bayesian mediation analysis that ensures to measure the extent to which—and by how much—the total effect of compulsory citizenship behaviors on the moral disengagement is accounted for by the mediation effect, that is anger toward organization (VanderWeele, 2013; Ananth, 2019). The corresponding absolute proportion mediated effect size was calculated as 33.74%. The ratio indicates that approximately 33.7% of the effect of the compulsory citizenship behaviors on moral disengagement was mediated through the nurses’ anger toward organization.

## Discussion

This paper validates that CCBs have the potency to explain the moral disengagement of nurses. This result emerges in line with the depletion of employee resources. More specifically, poor management and resourcing of nurses led to an intensification of their work and enforcement of extra-role behaviors. These extra-role behaviors, which nursing managers enforce, lead to nurses’ moral disengagement (Muraven et al., 1998). This result is consistent with proposition of Vigoda-Gadot (2006) that CCBs will lead to adverse outcomes.

Furthermore, our results demonstrated that CCBs influenced moral disengagement through the mediation of anger toward the organization. From the resource depletion theory lens (Kong and Drew, 2016), if extra-role behaviors are reluctantly fulfilled due to forcible actions of nursing managers or powerful others, they can cause a reduction and loss of resources for nurses. So, they may become emotionally activated (Vigoda-Gadot, 2007; Fida et al., 2015; Lüdecke et al., 2021) and feel anger toward the organization. Hence, negative emotions (such as anger, fear, and sadness) trigger moral disengagement by weakening moral self-regulation (Muraven and Baumeister, 2000; Fida et al., 2018). When negative emotions are experienced because of CCBs, individuals are inclined to behave short-term and impulsive. They hope to change their moods using energy to feel better (Christian and Ellis, 2011). However, the actions consume the limited strength essential for self-control (Muraven et al., 1998). Consequently, since a significant portion of this energy is consumed to regulate negative and aversive emotions (e.g., anger), employees become more prone to moral disengagement (Muraven and Baumeister, 2000).

COVID-19 has caused significant damage to health workers, especially to nurses’ mental and psychological health, such as psychological distress and moral injury (Hossain and Clatty, 2021). The risk of being infected with the virus, excessive workload, and isolating themselves from society and even from their families to prevent contamination, the nurses reduce their social support and cause stress on them to increase excessively (Zhang et al., 2020). These emerging stress factors cause many negative results, such as anger, anxiety, insomnia, and depression (Huang et al., 2020; Zhang et al., 2020). Anger and compulsivity are some of the consequences of COVID-19 (Huang et al., 2020; Huerta-González et al., 2021). Litz and Kerig (2019) emphasized that social, psychological, and spiritual distress can cause moral disengagement. Supporting this notion, moral disengagement could emerge as an initial reaction to anger or frustration (Jameton, 1993). Based on these perspectives, the interaction of the expectations of CCBs, caused by the poor management of workloads in the COVID-19 pandemic, led to negative consequences such as anger and moral disengagement in nurses.

Moral disengagement is an orientation that predisposes employees to counterproductive workplace behaviors (Hystad et al., 2014). Employees with moral disengagement have devastating consequences for organizations because they legitimize unethically (Barnes et al., 2011; Selart and Johansen, 2011) and deviant workplace behaviors (Christian and Ellis, 2011). In the long run, morally disengaged employees can damage co-workers’ productivity, threaten the organization’s well-being, lead to the breach of safety behaviors in the workplace, and even cause patient violence (Leidner et al., 2010; Caprara et al., 2014). Since negative behaviors generally undermine the goals and interests of an organization, it is critical to creating an organizational environment that will not allow these behaviors (Yildiz et al., 2022b). Additionally, the responsible and accountable leadership which does not coerce nurses to display CCBs (Podsakoff et al., 1990; Kim, 2014; Purwanto et al., 2021) but empowers and develops the nursing profession with adequate resources, could prove crucial in combatting the precarity experienced during the COVID-19 pandemic by this group of workers.

### Practical implications

The proportion-mediated ratio is a useful indicator for policy-relevant recommendations (VanderWeele, 2013). In other words, this indicator addresses the importance of the underlying factor (anger) in the relationship between independent (CCBs) and dependent variables (moral disengagement). Because the amount of proportion mediated was 33.74%, policymakers should consider this indicator by generating preventive solutions or policies against compulsory citizenship behaviors. In other words, this ratio explains that mechanisms other than anger may also play a role between CCB and moral disengagement. That is not to be deceived by the full mediating effect of anger in the frequentist approach; on the contrary, it emphasizes that it is necessary to focus on the idea that anger is insufficient to explain the relationship between CCBs and moral disengagement fully.

COVID-19 has caused excessive workloads, especially for healthcare professionals, compared to other occupational groups (Cheong et al., 2022). A meta-analytic study conducted on 36 healthcare workers recently found positive relationships (work engagement—job satisfaction) in all studies before COVID-19 turned negative during the COVID-19 process (Yildiz et al., 2022b). Söğütlü et al. (2021) also found that COVID-19 has caused an increase in anger, anxiety, insomnia, severity, and emotion regulation difficulty in healthcare workers. When these findings are evaluated, it can be said that COVID-19 has significant psychological and physical destructive effects, especially on healthcare workers. The exposure of health workers to CCBs has reached its peak during the COVID-19 process (Unaldi Baydin et al., 2020; Yildiz and Elibol, 2021). Considering the nature of CCBs and the excessive workload caused by the COVID-19 process, the expectation and pressure from employees to do things outside of their duties reached their peak. In this context, it is recommended that countries develop effective crisis management strategies in terms of combating decline and precarity in healthcare systems. For example, healthcare policy can train managers and leaders on how they can better resource and support nurses in their struggle with precariously intensified working conditions.

Additionally, policymakers can reduce the burden on health workers by investing in health tools and equipment that will minimize the workload on employees or by establishing the necessary technological infrastructure. Job design strategies can be developed to enable hospital administrators to rest the employees and distribute the entire burden equally on the employees. In addition to these, psychological support and wellness programs can be provided to increase the psychological resilience of health professionals to prevent moral disintegration because of the pressure and anger they have experienced, and the employees should easily access this support. As risk management strategies, health workers who will be needed in another possible pandemic can be planned with proactive strategies in advance, with real data-based simulations depending on the data in the pandemic. In addition, educational institutions and institutes should include the issue of combating the pandemic in the training programs of healthcare workers. In this way, the training of health workers who are equipped to take part in a possible pandemic will be done proactively.

Moreover, CCBs are a hidden threat and lead to many negative consequences, but human resource managers cannot easily notice them. Hospital managers should take protective measures to prevent CCBs because these behaviors have a spillover effect on other domains. Considering that the most fundamental factor leading to CCBs is workload, it is necessary to employ enough nurses first. If this is not done, the number of nurses per patient will increase, and managers will ask their employees to exhibit extra-role behaviors. Therefore, these non-role expectations will cause anger toward the organization and, in turn, moral disengagement. Also, as emotions are contagious (Robbins and Judge, 2013), negative emotions may decrease the quality of service, and even patients may be mistreated. Hence, to improve the emotional management skills of managers and nurses, emotional intelligence training programs should be included more in the nursing curriculum and on-the-job training programs (Gou et al., 2022).

Even if employees are forced to perform CCBs due to staff shortages in COVID-19 conditions, organizational justice should always be observed because employees can violate patient and workplace safety rules in an unfair workplace. Even in pandemic conditions, flexible working arrangements should be created by considering employees’ differences (Zhuang, 2021). Also, considering the buffering effects of high moral identity on moral disengagement (Detert et al., 2008; Zhang et al., 2019), moral identity can be used as a wise coping strategy so that anger does not turn into moral disengagement. At this point, health administrations or organizations should establish moral and ethical standards and adhere to them. It is another option for health institutions to employ employees with important moral characteristics.

### Theoretical implications

This study contributed to the existing literature by examining the effect of CCBs on moral disengagement through anger toward organizations considering the resource depletion theory. Second, this study reveals anger toward organizations as an underlying mechanism between CCBs and moral disengagement. This study makes a significant contribution by uncovering the direct link between CCBs and moral disengagement. When the CCBs literature is examined, most of the studies have built their models on perceptions (Wang and Huang, 2019; Chen et al., 2021), attitudes (Peng and Zhao, 2011; Che, 2015), and behaviors (Su et al., 2021; Yildiz and Elibol, 2021) related to CCB. However, an interesting gap is related to emotions such as anger (Che, 2015).

Emotions play a pivotal role in shaping decision-making, interaction, and cognitive processes (Sreeshakthy and Preethi, 2016). Considering that moral disengagement is a cognitive process (Bandura, 1999), the importance of emotions, especially anger, becomes more apparent. This study revealed the importance and role of anger as an emotion in the CCBs literature, which focuses on results such as productivity losses or behaviors. Our results show that nurses exposed to CCBs develop feelings of anger toward their organizations, and this situation causes the moral decision-making mechanisms of the employees to degenerate. This evidence demonstrates that exposure to CCBs causes emotions such as anger that deplete employees’ positive energy and resources. Still, it can cause employees’ moral decision-making mechanisms to remain unfulfilled. In other words, the extra behaviors imposed on the employees without volunteering have more harm than benefits to the organizations. Therefore, these behaviors have a structure that reduces the efficiency and energy of the employees both emotionally (anger) and cognitively (moral disengagement). One of the original contributions of this study is that employees forced to CCBs under extreme workload conditions arising from the COVID-19 process increase their emotions of anger and, accordingly, their moral reasoning systems degenerate. As Hossain and Caltty (2021, p. 23) express, the devastating effects of COVID-19 on nurses are “the overwhelming number of deaths, patients isolated and dying alone, and the ever-present fear of being infected and then infecting colleagues, family, and friends due to the lack of protective gear or known protocols takes its toll on emotional and psychological well-being. For nurses, the experience of this significant (hopefully once-in-a-lifetime) event can inflict ongoing moral injury.” Thus, the research findings were consistent with the findings of both resource depletion theory and previous studies (Huang et al., 2020; Bayrak et al., 2021; Hossain and Clatty, 2021; Söğütlü et al., 2021).

## Conclusion

This study offers a novel contribution about how CCBs lead to moral disengagement among Turkish nurses, mediated by anger toward organizations. The hypothesized research model is based on the resource depletion theory. The cross-sectional data were collected from nurses employed at private and public hospitals in Turkey using the snowball sampling method. Two hundred ninety-four valid questionnaires were analyzed by using CB-SEM and the Bayesian mediation analysis. The analyses revealed that compulsory citizenship behaviors only indirectly affect moral disengagement through anger toward the organizations. However, the proportion mediated takes a small part of the total effect, which it causes the issue of what are the other pathways through which the CCBs affect moral disengagement rather than a mediator variable.

### Limitations and future research

This research naturally has some limitations. First, since the cross-sectional design cannot be used to infer causality, it could be meaningful to test the model in a longitudinal research design. Second, the data were collected from Istanbul, Turkey; therefore, the generalization of the results for Turkey is limited. Third, due to its explorative nature, the main research focus of the researchers concentrated on the study variables. Another limitation of the study is that since this study was conducted during the COVID-19 period, the study’s results are limited to the situational conditions of an unusual specific period. Also, since there are not enough studies in the relevant literature to compare the results of the research, it is recommended to make a similar study for future researchers. It is recommended that future researchers carry out similar studies after the pandemic and compare them with the findings during the pandemic process to detect the devastating effects of the pandemic. Further research should include control variables to their models that may influence the constructs of this research. Because the proportion mediated explains 33.74% of the total effect, the full mediational model is not enough to explain the mechanism among the research variables. Other potential mediators, such as organizational commitment (Yildiz, 2016), job stress (Unaldi Baydin et al., 2020), psychological ownership (Vandewalle et al., 1995), and organizational resentment (Rice, 2013) should be considered by future research.

## Data availability statement

The raw data supporting the conclusions of this article will be made available by the authors, without undue reservation.

## Ethics statement

The studies involving human participants were reviewed and approved by the Committee on Ethics in Research on Humans of Istanbul University (Approval number: 2021-142). The patients/participants provided their written informed consent to participate in this study.

## Author contributions

BY, HY, and MO contributed to conception and design of the study and wrote sections of the manuscript. BY organized the database. BY and HY performed the statistical analysis and wrote the first draft of the manuscript. All authors contributed to the article and approved the submitted version.

## Conflict of interest

The authors declare that the research was conducted in the absence of any commercial or financial relationships that could be construed as a potential conflict of interest.

## Publisher’s note

All claims expressed in this article are solely those of the authors and do not necessarily represent those of their affiliated organizations, or those of the publisher, the editors and the reviewers. Any product that may be evaluated in this article, or claim that may be made by its manufacturer, is not guaranteed or endorsed by the publisher.
