# Supplementary material for: How do compulsory citizenship behaviors affect moral disengagement in organizations? Significance of anger toward the organization during the COVID-19 pandemic
Source: Front Psychol. 2022 Nov 25;13:1038860. doi: 10.3389/fpsyg.2022.1038860 (PMC9732554; doi:10.3389/fpsyg.2022.1038860)
Supplement: Supplementary file 1 [file Table_1.docx]

**Appendix: Scales in English and Turkish**

| **Scales in English** | **Scales in Turkish** |
| --- | --- |
| ***Compulsory citizenship behaviors scale*** | ***Zorunlu vatandaşlık davranışları*** |
| 1. The management in this hospital puts pressure on employees to engage in extra-role work activities beyond their formal job tasks. | 1. Çalıştığım hastanedeki yönetim, hemşirelerin mevcut görev tanımlarının ötesinde davranmaları için baskı yapmaktadır. |
| 2. There is social pressure in this hospital to work extra hours, beyond the formal workload and without any formal rewards. | 2. Çalıştığım hastanede herhangi bir karşılığı olmaksızın, hemşireler üzerinde olması gereken iş yükünün ötesinde fazladan çalışmaları için sosyal baskı vardır. |
| 3. I feel that I am expected to invest more effort in this job than I want to and beyond my formal job requirements. | 3. İşimde yasal iş gereklerim ve istediğim dışında fazla çaba harcamam yönünde bir beklenti olduğunu hissediyorum. |
| 4. I feel that I am forced to help other nurses beyond my formal obligations and even when I am short on time or energy. | 4. Yeterli zaman ve enerjim olmadığında dahi yasal zorunluluğum olmamasına rağmen diğer hemşirelere yardım etmeye zorlandığımı hissediyorum. |
| 5. I feel that I am forced to assist my supervisor against my will and beyond my formal job obligations. | 5. Özgür irademe karşı olmasına ve yasal zorunluluğum olmamasına rağmen yöneticime yardım etmeye zorlandığımı hissediyorum. |
| ***Anger toward organization scale*** | ***Örgüte yönelik öfke*** |
| 1. I feel angry toward my organization. | 1. Örgütüme karşı öfkeli hissediyorum. |
| 2. I feel irritated toward my organization. | 2. Örgütüme karşı sinirli hissediyorum. |
| 3. I feel irritated toward my organization. | 3. Örgütüme karşı kızgın hissediyorum. |
| ***Moral disengagement scale*** | ***Ahlaki kayıtsızlık*** |
| 1. It is okay to spread rumors to defend those you care about. | 1. Önem verdiğin kişileri savunmak için söylentiler yaymak sorun değil. |
| 2. Taking something without the owner’s permission is okay as long as you are just borrowing it. | 2. Ödünç aldığınız sürece sahibinin izni olmadan bir şey almanız sorun değildir. |
| 3. Considering the ways people grossly misrepresent themselves, it is hardly a sin to inflate your own credentials a bit. | 3. Başkalarının kendilerini büyük ölçüde yanlış tanıtma şekillerini göz önünde bulundurduğunuzda, kendinizi biraz abartmakta sorun yoktur. |
| 4. People should not be held accountable for doing questionable things when they were just doing what an authority figure told them to do. | 4. İnsanlar, otorite sahibi birinin direktiflerini yerine getirirken, yaptıkları sorgulanabilir şeylerden sorumlu tutulmamalıdır. |
| 5. People cannot be blamed for doing things that are technically wrong when all their friends are doing it too. | 5. İnsanlar eğer tüm arkadaşlarının teknik olarak yanlış yaptığı bir şeyi yapıyorsa yanlış yaptıkları şeyler için suçlanmamalıdır. |
| 6. Taking personal credit for ideas that were not your own is no big deal. | 6. Başkalarına ait fikirlerden itibar kazanmak sorun değildir. |
| 7. Some people have to be treated roughly because they lack feelings that can be hurt. | 7. İncinebilecek hislerden yoksun oldukları için bazı insanlara kaba davranılması gerekir. |
| 8. People who get mistreated have usually done something to bring it on themselves | 8. Kötü muameleye maruz kalan insanlar genellikle bunu hakkedecek bir şeyler yapmışlardır. |
